# Albumin-Modified Melanin-Silica Hybrid Nanoparticles Target Breast Cancer Cells via a SPARC-Dependent Mechanism

**DOI:** 10.3389/fbioe.2020.00765

**Published:** 2020-07-08

**Authors:** Gennaro Sanità, Paolo Armanetti, Brigida Silvestri, Barbara Carrese, Gaetano Calì, Giulio Pota, Alessandro Pezzella, Marco d’Ischia, Giuseppina Luciani, Luca Menichetti, Annalisa Lamberti

**Affiliations:** ^1^Department of Molecular Medicine and Medical Biotechnology, University of Naples Federico II, Naples, Italy; ^2^Institute of Clinical Physiology, National Research Council, Pisa, Italy; ^3^Department of Chemical, Materials and Production Engineering, University of Naples Federico II, Naples, Italy; ^4^Institute of Endocrinology and Molecular Oncology, National Research Council, Naples, Italy; ^5^Department of Chemical Sciences, University of Naples Federico II, Naples, Italy

**Keywords:** albumin, SPARC, nanoparticles, photoacoustic, melanin

## Abstract

Bioconjugation of a recently developed photoacoustic nanoprobe, based on silica-templated eumelanin-silver hybrid nanoparticles (MelaSil_Ag-NPs), with human serum albumin (HSA) is disclosed herein as an efficient and practical strategy to improve photostability and to perform SPARC mediated internalization in breast cancer cells. Modification of NPs with HSA induced a slight viability decrease in breast cancer cells (HS578T) and normal breast cells (MCF10a) when incubated with HSA-NPs up to 100 μg/mL concentration for 72 h and a complete suppression of hemotoxicity for long incubation times. Uptake experiments with MelaSil_Ag-HSA NPs indicated very high and selective internalization via SPARC in HS578T (SPARC positive cells) but not in MCF10a (SPARC negative cells), as evaluated by using endocytosis inhibitors. The binding of SPARC to HSA was confirmed by Co-IP and Dot-blot assays. Additional studies were performed to analyze the interaction of MelaSil_Ag-HSA NPs with protein corona. Data showed a dramatic diminution of interacting proteins in HSA conjugated NPs compared to bare NPs. HSA-coated MelaSil_Ag-NPs are thus disclosed as a novel functional nanohybrid for potential photoacoustic imaging applications.

## Introduction

Nanotechnology in cancer disease is entirely dedicated to the study of the therapeutic drugs and/or imaging agents delivery. A high biocompatibility and efficient delivery properties of nanoparticles (NPs) were recognized as important targets toward multipurpose efficient nanomedical solution since ideal carrier properties include safety and optimal bioavailability (Naahidi et al., 2013; Adabi et al., 2017). Primary requirements for NPs include stability, nontoxicity, non-immunogenicity and targeting capability to a specific site. Most of these properties can be achieved and tuned via clever manipulation of their shape, size and surface chemistry, which are responsible for cellular internalization, half-life in blood and immune response (Albanese et al., 2012; Shahbazi et al., 2013; La-Beck and Gabizon, 2017; Sukhanova et al., 2018). A valuable option includes functionalization of the NPs surface with natural biomolecules that specifically recognize cancer cells via receptors abundantly present on the surface of tumor cells or overexpressed in stroma (Jahan et al., 2017; Muhamad et al., 2018; Zhang et al., 2018). Furthermore, the presence of a biomolecule on the nanoparticle surface can improve stability, nontoxicity, non-immunogenicity and bioavailability.

A potential candidate for NPs stabilization in the field of nanomedicine and cancer therapy is albumin, which has been successfully applied in Abraxane (Von Hoff et al., 2011; Yardley, 2013; Al-Batran et al., 2014). It is the most abundant plasma protein in human blood with a molecular weight of 66.5 kDa and a molecular size of ∼7.2 nm and has been already explored as a useful medical device for therapeutic and diagnostic agents delivery, due to its non-immunogenicity and nontoxicity (He and Carter, 1992; Bairagi et al., 2015; Mehtala et al., 2015). Moreover, albumin interacts with cellular receptors that include glycoproteins Gp60, Gp30, and Gp18 and the secreted protein acidic and rich in cysteine (SPARC), and these interactions can be used for a specific targeted delivery of NPs (Schnitzer, 1992; Tiruppathi et al., 1996; Desai et al., 2009; Chlenski et al., 2016). Particular interest among albumin binding proteins is attracted by SPARC, which is overexpressed in the extracellular matrix and in various cancer cells and is associated with tissue growth and cell proliferation. It acts as the major transmembrane receptor for albumin and its overexpression can enhance cellular internalization of the albumin nanoparticles. It has been supposed that the tumor increase uptake of nab-paclitaxel (Abraxane) occurs by this SPARC mediated pathway (Yardley, 2013; Al-Batran et al., 2014; Vaz et al., 2015).

Recently, a biocompatible hybrid organo-inorganic nanoprobe for Photoacoustic imaging (PAI) was designed and synthesized by in-situ generation of a eumelanin-silver hybrid via redox interactions in a silica templating matrix. Self-assembly into multi-cluster architectures of Ag in a hybrid melanin-silica phase resulted in an enhanced photoacoustic contrast of great promise for PAI application. Furthermore, the hybrid phase is a versatile chemical platform which can be easily functionalized with a large set of bioactive molecules (Silvestri et al., 2019).

Photoacoustic imaging is an exciting new modality that uses non-ionizing radiation for real-time imaging with high spatial resolution and penetration depth (Mallidi et al., 2011). Nanoparticle-based contrast agents are important tools in photoacoustic imaging thanks to their strong and stable signal and to the possibility to target specific cell populations (Lemaster and Jokerst, 2017). Furthermore, PAI could be easily combined with poor invasive cancer therapies, such as photothermal ablation (PTA) (Liu et al., 2016, 2018; Fan et al., 2019).

Prompted by the promising perspectives derived from combining MelaSil_Ag NPs with a biomodulator for improving cell internalization and targeting, a systematic study was undertaken to explore the potential of human serum albumin (HSA) to modify the NPs to target breast cancer cells. Herein, the high biocompatibility and hemocompatibility properties of the HSA-modified NPs are reported along with the very strong increase in signal stability and the efficient and specific uptake by a cancer cell line via a SPARC-mediated mechanism.

## Materials and Methods

### Reagents

N-(3-dimethylaminopropyl)-N’-ethyl-carbodiimide hydrochlo ride (EDC), N-hydroxysuccinimide (NHS), 3-(amino propyl)triethoxysilane (APTES), Ethanol (absolute, ≥99.8%), Rhodamine B isothiocyanate (RBITC), Methanol (absolute, ≥99.8%), Fluorescamine, 2-(N-morpholino) ethanesulfonic acid (MES), Sodium Chloride (NaCl), recombinant Human Serum Albumin (rHSA), Chlorpromazine hydrochloride, Genistein Synthetic were purchased from Sigma-Aldrich (St Louis, MO, United States). Phosphate-buffered saline (PBS) was purchased from Gibco (Grand Island, NY, United States). Live Cell Labeling Kit CytoPainter was purchased from ABCAM (Cambridge, United Kingdom). CellTiter-Glo^®^ Luminescent Cell Viability Assay was purchased from Promega (Madison, WI, United States). Rabbit Anti-SPARC monoclonal antibody was purchased from ABCAM (Cambridge, United Kingdom). Mouse Anti-HSA monoclonal antibody was purchased from Fitzgerald Industries International (North Acton, MA, United States).

### HSA MelaSil_Ag NPs Conjugation

MelaSil_Ag nanoparticles were collected by centrifugation, washed three times with absolute ethanol and aminosilanized by incubation with a 5% (v/v) APTES solution in absolute ethanol at room temperature (RT) for 1 h, to form a thin silane film on their surface. The amino groups of silanized NPs were covalently conjugated to the carboxyl groups of rHSA via NHS/EDC chemistry. Briefly, MelaSil_Ag-NPs (2.0 mg) were incubated with 2 mg of rHSA in the presence of EDC (20 mM)/NHS (30 mM) in 0,1 M MES/0.5 M NaCl buffer pH 6.0, to promote the reaction (ON at RT, under stirring). After the reaction, NPs suspension was centrifuged, and the pellet was collected and washed twice with the reaction buffer and then with PBS 1x. To test the efficiency of the chemical modifications, fluorescamine assay was used. This reagent reacts with primary amines to form highly fluorescent products. To this aim, APTES and rHSA modified NPs were incubated with fluorescamine 0.5 mM in phosphate buffer 20 mM pH 7 for 30 min, under stirring in the dark. After two washing, fluorescence intensity was measured with a spectrofluorometer (Ex 366 nm/Em 488 nm).

### MelaSil_Ag-HSA NPs Dot Blot Analysis

20 μg of MelaSil_Ag and MelaSil_Ag-HSA NPs were spotted on the nitrocellulose membrane. Next, the membrane was incubated with an anti-HSA primary antibody for 18 h, and, successively, a secondary antibody conjugated with horseradish peroxidase (HRP). After 3 washes, membrane was developed using enhanced chemiluminescence detection reagents and exposed to X-ray film. This analysis was also used to evaluate the stability of the rHSA bond to NPs surface. To this aim, MelaSil_Ag-HSA NPs were incubated in cell culture medium at 100 μg/ml for different times (1, 6, 24, 48, 72, and 96 h) at 37°C. NPs (20 μg) were collected, washed three times with PBS 1x and spotted on nitrocellulose membrane. Next, the membrane was incubated with anti-HSA primary antibody for 18 h, developed using enhanced chemiluminescence detection reagents and exposed to X-ray film.

### Dynamic Light Scattering (DLS) and ζ-Potential Characterization

Size distribution and ζ-potential were measured by a Zetasizer (Nanoseries, Malvern) using the laser dynamic scattering (λ = 632.8 nm) and the particle electrophoresis techniques, respectively. All the samples were diluted up to a droplet concentration of approximately 0.025% w/v by using Milli-Q water. A detecting angle of 173° and 5 runs for each measurement (1 run lasting 100 s) were used in the calculations of the particle size distribution. ζ-potential analyses were carried out by setting 50 runs for each measurement.

### Transmission Electron Microscopy (TEM)

Transmission electron microscopy analysis was carried out with about 10 μl of solution containing NPs (5 μg) spread on a copper grid (200 mesh with carbon membrane). TEM images were obtained using a TECNAI 20 G2: FEI Company with a camera Eagle 2HS. The images were acquired at 200 kV; camera exposure time: 1 s; size 2048 × 2048.

### Fourier-Transform Infrared Spectroscopy

Chemical composition of HSA-functionalized MelaSil_Ag nanoparticles was analyzed by Fourier-transform infrared (FTIR) spectroscopy. Spectra were recorded by a Thermo-Nicholet NEXUS Continuum XL (Thermo Scientific, Waltham, MA, United States) equipped with a microscope, on dried NPs samples. The spectrum of bare NPs was used as control. Spectra were collected in the range of 4000–600 cm^–1^ with a resolution of 16 cm^–1^.

### Protein Corona Analysis

To study corona proteins bonded to HSA functionalized and bare NPs surface, both types of nanoparticles (100 μg) were incubated in DMEM with FBS 10% (v/v) at 37°C for 0.5, 1, 6, 24, 48, and 72 h. After 3 washes in PBS 1×, NPs were resuspended in Leammli Buffer 1× (4% SDS w/v, 20 % glycerol v/v, 1.43 M 2-mercaptoethanol, 0.004 % bromophenol blue w/v, UREA 2M in 0.125 M Tris–HCl pH 6.8) incubated for 18 h at RT, heated at 95°C and analyzed by 12% polyacrylamide SDS-PAGE.

### Cell Culture

Human breast carcinoma cell line (HS578T) and mammary breast fibrocystic disease cell line (MCF10a), obtained from the American Type Tissue Collection (Rockville, MD, United States), were grown in DMEM (GIBCO) and MEGM (Lonza), respectively. DMEM was supplemented with 10% heat inactivated FBS (GIBCO), 100 U/ml penicillin, 100 mg/ml streptomycin, and 1% L-glutamine. MEGM was supplemented with Mammary Epithelial Cell Growth Medium Bullet Kit (Lonza),100 nM cholera toxin (Sigma Aldrich) and 5% heat inactivated FHS (Lonza). All cell lines were grown at 37°C in a 5% CO_2_ atmosphere.

### Cytosolic Extracts and Western Blot Analysis

HS578T and MCF10a cells were scraped, washed twice in 1 × PBS and resuspended in 20 – 40 μl of modified RIPA buffer (Arcucci et al., 2014) for 30 min on ice, centrifuged at 14,000 × *g* for 30 min at 4°C and supernatant, containing proteins, was recovered. For cell culture medium analysis, cells were incubated in 96 multiwell plate for 24 h. The culture medium was collected and centrifugated at 3000 rpm for 10 min to remove cells and debris. Protein concentration was determined by Bradford method, using the Bio-Rad protein assay and compared with BSA standard curve. For the western blot evaluation, 20 μg of proteins from cell culture medium and cytosolic extracts were used. Proteins were separated by 12% SDS-PAGE, electro-transferred to PVDF membrane and reacted with the different antibodies. Blots were then developed using enhanced chemiluminescence detection reagents (Western Bright ECL, Advansta) and exposed to X-ray film.

### Cell Viability Assays

For Cell-Titer GLO assay, cells were seeded into 96-well microtiter plates (BD Falcon, United States) at the density of 10 × 10^3^ cells/well and incubated with MelaSil_Ag NPs and MelaSil_Ag-HSA NPs at increasing concentrations (25, 50, and 100 μg/ml) in triplicate. The assay was performed after 24 h, 48 h and 72 h of incubation, according to the manufacturer’s instructions. Luminescence was recorded for 0.25 s per well by Multilabel Reader (PerkinElmer, Waltham, MA, United States).

For CytoPainter Live Cell assay, cells were seeded into 24-well microtiter plates at the density of 40 × 10^3^ cells/well and incubated with MelaSil_Ag NPs and MelaSil_Ag-HSA NPs at 100 μg/ml. The assay was performed after 24, 48, and 72 h of incubation, according to the manufacturer’s instructions. After incubation, cells were observed by fluorescence microscopy.

### Hemotoxicity Assay

Heparin-stabilized fresh blood samples were obtained from healthy human volunteers and used within 1 h. After dilution with PBS 1×, red blood cells (RBCs) were isolated from serum by centrifugation (2,000 × *g*, 6 min). After five washing with PBS 1×, RBCs were diluted 1:20 to obtain 5% hematocrit. Subsequently, RBCs suspension was mixed with increasing concentration (50 and 100 μg/ml) of bare or rHSA functionalized NPs. The mixtures were then vortexed and incubated for 1, 4, 8, 24, and 72 h at room temperature in static conditions. After these times, the samples were gently vortexed and centrifuged (9,000 × *g*, 3 min). PBS and water were used as negative and positive controls, respectively. The obtained supernatants were evaluated by naked eye and spectrophotometric analysis at 577 nm.

### Confocal Microscopy

Cells (10 × 10^3^ or 5 × 10^3^/coverslip) were plated on 10 mm glass coverslips placed on the bottom of 24-well plate, allowed to attach for 24 h under normal cell culture conditions and then incubated with MelaSil_Ag^∗^-HSA NPs at concentrations of 50 μg/ml for 18 h at 37°C. Cells were washed with PBS, fixed in 2% formaldehyde for 10 min, washed 3 times with PBS, and stained with membrane stain WGA-Alexa Fluor 488 Conjugate (Invitrogen, Carlsbad, CA, United States) according to the manufacturer’s instructions. Cell nuclei were then stained with Hoechst 33342 (Invitrogen, Carlsbad, CA, United States). Cells were then spotted on microscope slides and analyzed. Experiments were carried out on an inverted and motorized microscope (Axio Observer Z.1) equipped with a 63×/1.4 Plan-Apochromat objective. The attached laser-scanning unit (LSM 700 4× pigtailed laser 405–488–555–639; Zeiss, Jena, Germany) enabled confocal imaging. For excitation, 405, 488, and 555 nm lasers were used. Fluorescence emission was revealed by Main Dichroic Beam Splitter and Variable Secondary Dichroic Beam Splitter. Triple staining fluorescence images were acquired separately using ZEN 2012 software in the red, green and blue channels at a sampling of 512 × 512 pixels, with the confocal pinhole set to one Airy unit and then saved in TIFF format.

### Immunoprecipitation

10 and 50 μg of rHSA were added to HS578T cytosolic extracts (500 μg). Then, the samples were immunoprecipitated with 4 μg of anti-SPARC antibody, for 16 h at 4°C. Immune complexes were collected with 20 μl of protein G-agarose for 2 h at 4°C. The protein G-agarose/immune complex was washed four times with cold PBS 1×, resuspended in 30 μl of loading buffer, heated to 95°C for 5 min and used for Western blot analysis by using anti- HSA antibody.

### MelaSil_Ag-HSA NPs-SPARC Interaction

MelaSil_Ag-HSA NPs (100 μg) were incubated in HS578T conditioned medium at 37°C under stirring for 24 h. NPs were collected, washed three times with PBS 1x, resuspended in Leammli Buffer 1x, incubated for 18 h at RT and heated at 95°C. Proteins were resolved by SDS-PAGE and analyzed by western blot with anti- SPARC antibody.

### Cytofluorimetric Analysis

HS578T and MCF10a cell lines were incubated with MelaSil_Ag^∗^-HSA NPs at increasing concentrations for different times at 37°C. HS578T cells were also incubated with MelaSil_Ag^∗^ at different concentrations for 18 h. After three washes in PBS 1×, cells were resuspended in PBS 1× and analyzed with a Becton Dickinson FACScan flow cytometer. All measurements were carried out in triplicate, in three independent experiments. Cytofluorimetric analysis was also carried out in the presence of chlorpromazine (CPZ) and genistein (GEN). To this aim, HS578T cells were preincubated for 30 min with chlorpromazine (20 μg/ml) and with genistein (80 μg/ml) in PBS 1×. After 2 washes, MelaSil_Ag-HSA NPs (100 μg/ml) were added for 6 and 18 h.

### PA Experimentation in Test-Object Phantom

For all photoacoustic (PA) tests, the multi-modality imaging platform Vevo LAZR-X (FUJIFILM VisualSonics Inc.) was used. To check the specific spectral behavior and photostability were performed: the PA spectral analysis in the optical windows between 680 and 970 nm, and prolonged laser illumination at 705 nm over time. First, the experimentation was performed in a custom made phantom (Armanetti et al., 2018) consisting in a polypropylene box with inserted a series of coplanar polyethylene tubes (i.d = 580 μm, o.d. = 990 μm) in which the nanoparticles were loaded (Figure 8). Then, the phantom was filled and coupled with PA probe (Figure 8) with water. The temperature was kept in a physiological range between 30° and 40°C, controlled by an infrared thermometer. Around 40–50 μl of nanoparticles were loaded in each tube. The PA signal of MilliQ water was chosen as background.

### PA Assessment in Biological *ex vivo* Tissue

Once checked their performance in test-object analysis, the next step was performed by the *ex vivo* experimental tests, where samples of chicken breast were used as a biological matrix. A bolus of about 100 μl of NPs was injected. The samples were embedded in agarose matrix (∼1%) to keep the anatomical geometry fixed, then maintained at physiological temperature for the whole duration of PA acquisitions. We studied the signal provided from two different region of interest: the region of injection and another far to the injection site.

### Evaluation of PA Signal From Cells

After this, to evaluate the PA signal of cells incubated with NPs, HS578T cells were seeded into 24 multiwell plates, let growth for 24 h and incubated with MelaSil_Ag- HSA NPs at 100 μg/ml. After 18 h of incubation, cells were collected, washed with PBS 1× for three times and fixed with PFA 2% for 5 min at room temperature. After fixation, cells were washed with PBS 1x for three times, collected by centrifugation and the pellet was embedded in agarose matrix (∼1% w/v). Hence, this pellet was inserted in a cave cylindrical agar phantom, then fixed inside a box (Figure 10).

### Statistical Analysis

Results of the assays are expressed as mean ± SD of three independent experiments. Data are reported as average and SD. The statistical significance of differences among groups was evaluated using analysis of variance, using the software GraphPhad 8.0. The significance was accepted at the confidence level of 95% (*P* < 0.05).

## Results and Discussion

### Preparation and Characterization of HSA-Modified MelaSil_Ag NPs

**SCHEME 1:**
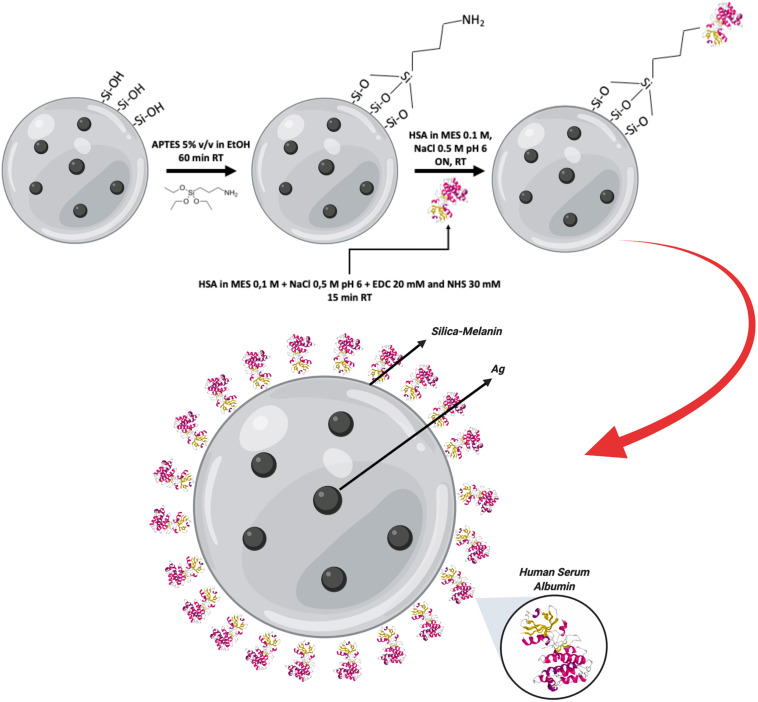
MelaSil_Ag-HSA NPs bioconjugation scheme. Workflow of MelaSil_Ag functionalization with HSA by EDC/NHS chemistry. NPs, nanoparticles; HSA, human serum albumin; EDC, 1-Ethyl-3-(3- dimethylaminopropyl)carbodiimide; NHS, N-Hydroxysuccinimide; MES, 2-(N-morpholino)ethanesulfonic acid.

MelaSil_Ag NPs (Silvestri et al., 2019) were conjugated with recombinant human serum albumin (rHSA) in order to improve their physicochemical and biological properties, such as biocompatibility and cellular internalization, since HSA is a promising material to produce nanoparticles for bioimaging and drug delivery (Mehtala et al., 2015; Nairi et al., 2018; Zhang et al., 2018). The bioconjugation was performed via silanization with APTES solution, which introduces highly reactive amino groups (–NH_2_) on the NPs surface available to covalent conjugation with the carboxyl groups of HSA by means of EDC/NHS chemistry (Scheme 1). The chemical modifications were evaluated by using Fluorescamine, which reacts with primary amines to form highly fluorescent products. As reported in Supplementary Table S1, the fluorescence intensity of HSA modified NPs was fivefold that of aminosilanized NPs, indicating the presence of more amino groups due to the presence of HSA.

Nanoparticles HSA-bioconjugation was also evaluated by FTIR spectroscopy. The comparison between FTIR spectra of bare nanoparticles and HSA functionalized NPs is reported in Supplementary Figure S1. The main characteristic bands of silica phase are clearly visible in MelaSil_Ag spectrum. The band at 1100 cm^–1^ was related to Si–O–Si stretching vibration modes in SiO_4_ units, while the bands at 950 cm^–1^ and 800 cm^–1^ were attributed to non-bridging Si–O stretching vibration and to Si–O–Si stretching vibration between two adjacent tetrahedral, respectively. The band at ∼3500 cm^–1^ was assigned to OH stretching vibration of surface silanol groups and absorbed water, while the band at 1640 cm^–1^ was attributed to bending vibration of free H_2_O. The MelaSil_Ag-HSA spectrum revealed new band at 2950 cm^–1^ attributed to C-H symmetric and asymmetric stretching vibrations of alkyl structures; additional intense bands are also visible at 1660, 1540 cm^–1^ related to stretching vibration of amide I and amide II, respectively. The band at 1420 cm^–1^ was attributed to the combination of the C-O stretching and O-H deformation vibrations and the band at 840 cm^–1^ related to C-H vibrations out of the plane of aromatic ring was also visible, confirming the presence of HSA in MelaSil_Ag-HSA sample (Grdadolnik and Maréchal, 2001; Socrates, 2001).

The binding of albumin to MelaSil_Ag NPs was further confirmed by dot blotting. Bare MelaSil_Ag NPs and MelaSil_Ag-HSA NPs spotted on nitrocellulose membrane were probed with a specific anti-HSA antibody and a secondary antibody conjugated to HRP. No chemiluminescent signal was detected for bare NPs while MelaSil_Ag-HSA NPs showed a chemiluminescent signal (Supplementary Figure S2). This result clearly proves the surface modification by HSA.

Moreover, also the stability of the HSA bioconjugation was evaluated by dot blot assay by using MelaSil_Ag-HSA NPs incubated in culture medium up to 96 h (Supplementary Figure S3). The result clearly showed the stability of the HSA bond on NPs surface, since no significant variation in HSA amount was observed, also for extended times. This data endorses that a covalent bond between ligands and nanoparticles is more stable and reproducible if compared to non-covalent conjugations, based on weak interactions (Nobs et al., 2004).

The NPs physicochemical properties were characterized before and after the surface modification by DLS, analyzing the hydrodynamic diameter, the polydispersity index (PDI), and the surface charge (ζ-potential) of the particles. An increase of the particles size from 190 ± 3.5 (PDI 0.20) to 230 ± 4.0 nm (PDI 0.24) after HSA conjugation was observed. This result is in excellent agreement with the presence of HSA on the nanoparticles surface. The bare and HSA-conjugated MelaSil_Ag-NPs exhibited ζ-potentials of −22 ± 1.3 mV and −25 ± 1.7 mV, respectively (Table 1). A value of −25 ± 1.7 mV showed by MelaSil_Ag-HSA NPs is in agreement with the isoelectric point of the protein (Vlasova and Saletsky, 2009).

**TABLE 1:** DLS and ζ-potential of MelaSil_Ag NPs before and after HSA bioconjugation.

	*R_*H*_ (nm)*	*ζ-potential (mV)*
*MelaSil_Ag*	190 ± 3.5	−22 ± 1.3
*MelaSil_Ag-HSA*	230 ± 4	−25 ± 1.7

*NPs, nanoparticles; HSA, human serum albumin, DLS, dynamic light scattering.*

**FIGURE 1:**
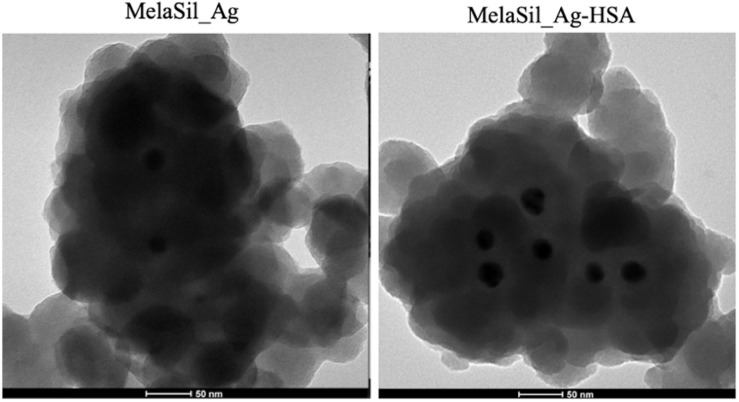
Transmission Electron Microscopy of MelaSil_Ag and MelaSil_Ag-HSA NPs.

The morphology of both MelaSil_Ag and MelaSil_Ag-HSA nanoparticles was investigated by TEM analysis and reported in Figure 1. The hybrid NPs consist of cluster architectures of small Ag NPs of ∼30 nm in diameter surrounded by a hybrid MelaSil phase. The whole nanoparticles size was ∼200 nm in diameters, in agreement with DLS investigation, and no separation between the melanin and silica phase was visible, suggesting an intimate mixing between the two components. Furthermore, an almost similar morphology was observed in MelaSil_Ag-HSA nanoparticles.

### Effect of HSA on Non-specific Binding of Serum Proteins

Nanoparticles can establish significant surface interactions with plasma proteins via hydrophobic or electrostatic bonds and can be surrounded by a protein corona. This corona influences physicochemical properties such as surface composition, surface charge, size, behavior and biological identity of the nanoparticles and can thus affect NPs toxicity and targeting/internalization capabilities (Gao and He, 2014; Corbo et al., 2016; Nguyen and Lee, 2017).

In order to identify the adsorbed proteins on MelaSil_Ag-HSA NPs, bare NPs and HSA-NPs were incubated in culture medium supplemented with 10% FBS for 0.5, 1, 6, 24, 48, and 72 h. The nanoparticles were then isolated, and the adsorbed proteins were separated by gel electrophoresis. As shown in Supplementary Figure S4, in bare NPs there are many interacting proteins between 245 and 20 kD up to 48 h of incubation, while most of these disappear at 72 h where a band of about 65 kD is still evident. Changes in protein profile, at different incubation times, was probably due to the evolution during time of the protein corona on NPs surface (Walkey and Chan, 2012; Branda et al., 2014; Hadjidemetriou and Kostarelos, 2017). Conversely, in HSA-modified NPs incubated in the same experimental conditions, only one band of about 65 kD (probably bovine serum albumin) was well detectable (Supplementary Figure S4; Walkey and Chan, 2012). This data suggests that the HSA modification inhibits the interaction of many serum proteins with the nanoparticle surface that could affect the NPs properties and behavior (Hoang et al., 2015).

### SPARC Protein Expression Levels Analysis by Western Blot

SPARC is a secreted protein that binds albumin (albumin-binding protein) and is highly expressed in many cancers. Some studies have proposed the involvement of SPARC in the uptake of albumin in tumors (Frei, 2011; Liu et al., 2015; Chlenski et al., 2016; Park et al., 2019) and in NPs internalization via HSA in cancer cells (Hoang et al., 2015). Thanks to this feature, SPARC represents a good molecular target for the active NPs delivery.

SPARC expression levels was investigated in a breast carcinoma cell line SPARC positive (HS578T) and in a mammary breast fibrocystic disease cell line SPARC negative (MCF10a) (Dan Theodorescu, 2013; Zhu et al., 2016) by western blot analysis by using a specific anti-SPARC antibody.

The investigation was carried out with total protein extracts and 24 h-conditioned medium of both cell lines. The results showed that very high level of SPARC protein was detectable in HS578T cells and in its conditioned medium. Conversely, no SPARC expression was revealed in MCF10a proteins extracts or conditioned medium (Supplementary Figure S5). Based on this observation, these two cell lines were chosen as experimental model to investigate the HSA-MelaSil_Ag NPs cell viability and internalization.

### *In vitro* Viability Assay

**FIGURE 2:**
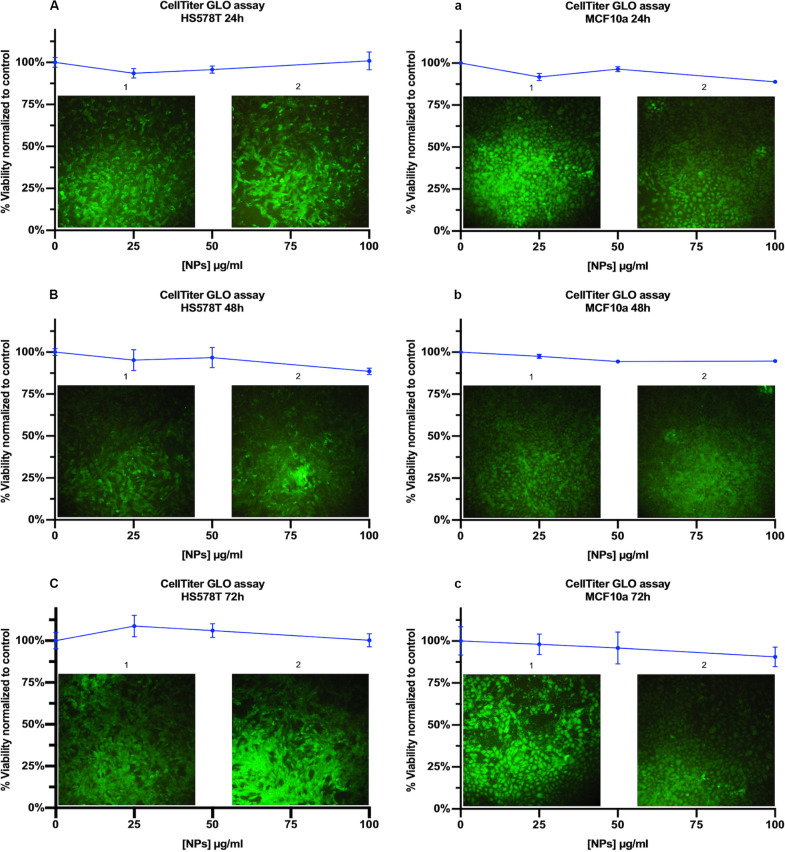
Cell viability assay. Cell-Titer GLO assay, of HS578T cells treated for **(A)** 24 h, **(B)** 48 h and **(C)** 72 h and MCF10a cells treated for **(a)** 24 h, **(b)** 48 h, and **(c)** 72 h with MelaSil_Ag-HSA NPs (25, 50, and 100 μg/mL). Insets are representative images of Calcein-AM fluorescent morphology images of HS578T and MCF10A cells before (1) and after (2) treatment with MelaSil_Ag NPs (50 μg/ml).

A critical issue for biomedical applications of new nanocarriers for contrast agents/drugs delivery is the evaluation of their potential toxicity and biocompatibility (Love et al., 2012). To evaluate the effect of HSA functionalization on NPs cytotoxicity, previously observed with bare NPs (Silvestri et al., 2019), two viability assays based on metabolic activity were chosen (Cell-Titer GLO and CytoPainter Live Cell). For Cell-Titer GLO, HS578T, and MCF10a cell lines were incubated in the presence of MelaSil_Ag-HSA NPs at increasing concentrations for 24, 48, and 72 h (Figure 2). For CytoPainter Live Cell, cells were incubated with HSA-conjugated NPs at 100 μg/ml for 24, 48, and 72 h (Figure 2 insets 1 control cells, insets 2 treated cells). The choice of the incubation period reflects the fact that the uptake was relatively rapid and had already occurred after 1 h; therefore, toxicity would be expected to be observed within 24 h (Silvestri et al., 2017). The doses of tested nanoparticles were similar to those causing good tolerance when administered in mice bloodstream (Varna et al., 2012; Martucci et al., 2016). The results clearly showed absence of toxicity of HSA-NPs if compared to bare NPs (Silvestri et al., 2019). This data could be due to HSA protective effect on MelaSil_Ag-HSA NPs, in fact the increase of NPs stability reduces the degradation and the release of metallic silver. In addition, no dependency on the exposure time and concentration was observed for the cytotoxicity of the HSA biofunctionalized nanoparticles. These results indicate an increase in the NPs biocompatibility after HSA biomodification.

### Effect of HSA Functionalization on NPs Hemotoxicity

The lysis of red blood cells (RBCs) in response to nanoparticles treatment is a measure of both membrane disruption and extreme cellular toxicity (i.e., necrosis) and is especially important for nanoparticles that are intended to be directly introduced into the bloodstream (Slowing et al., 2009; Mocan, 2013; Shahbazi et al., 2013). Therefore, the hemotoxicity evaluation is a critical preclinical analysis to assess the level of the NPs hemocompatibility, in order to prevent serious side effects (Terracciano et al., 2015).

To evaluate NPs hemotoxicity, quantitative and qualitative assay was performed by naked eye color evaluation and spectrophotometric analysis of the RBCs’ supernatant, respectively. To this aim, human RBCs were incubated with bare and HSA modified NPs (50 and 100 μg/ml) at increasing incubation times (1, 4, 6, 24, and 72 h). As positive and negative controls milliQ water and PBS 1x were used, respectively. Results showed a high degree of hemolysis, already after 1 h of incubation, when bare NPs were used. This effect is due to the presence of silanol groups on nanoparticles surface. These groups are highly reactive and can strongly interact with the positive charge and with the tetra-alkyl ammonium groups existent on the outer membrane surface of the RBCs, resulting in hemolysis (Paula et al., 2012; Ferenc et al., 2015; Martinez et al., 2015).

**FIGURE 3:**
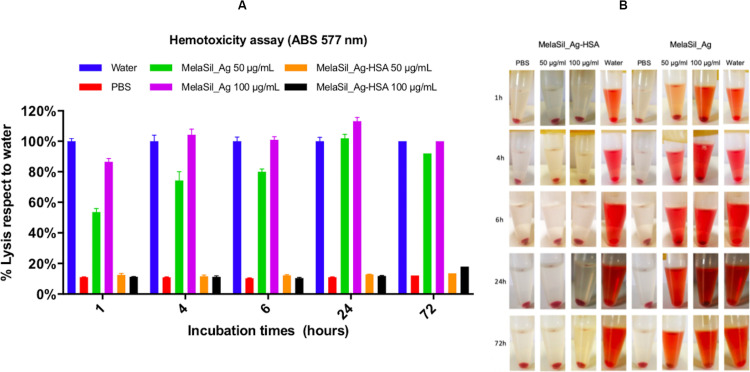
**(A)** Quantitative hemotoxicity evaluation by spectrophotometric analysis of MelaSil_Ag and MelaSil_Ag-HSA NPs. Measure of MelaSil_Ag and MelaSil_Ag- HSA hemotoxicity by assessing hemoglobin release by RBCs after incubation with MelaSil_Ag and MelaSil_Ag-HSA NPs at 50 and 100 μg/mL for 1, 4, 6, 24, and 72 h. **(B)** Qualitative hemotoxicity evaluation by naked eye analysis of MelaSil_Ag and MelaSil_Ag-HSA NPs. Representative images of naked eye evaluation of MelaSil_Ag and MelaSil_Ag-HSA NPs hemotoxicity by assessing hemoglobin release by RBCs after incubation with NPs at 50 and 100 μg/mL for 1, 4, 6, 24, and 72 h. NPs, nanoparticles; HSA, human serum albumin; RBCs, red blood cells.

Conversely, no hemolysis was observed when HSA-NPs were used, as shown in Figures 3A,B. The absence of NPs hemotoxicity after HSA surface modification shows that the hemocompatibility is due to the disappearance of the Si-OH groups and to the presence of HSA, which is known to be highly biocompatible since it is an endogenous protein. These results are in agreement with the viability data and suggest that MelaSil_Ag-HSA NPs could be used as contrast agent for long incubation times and at high concentrations.

### Uptake of Fluorescent MelaSil_Ag-HSA NPs

**FIGURE 4:**
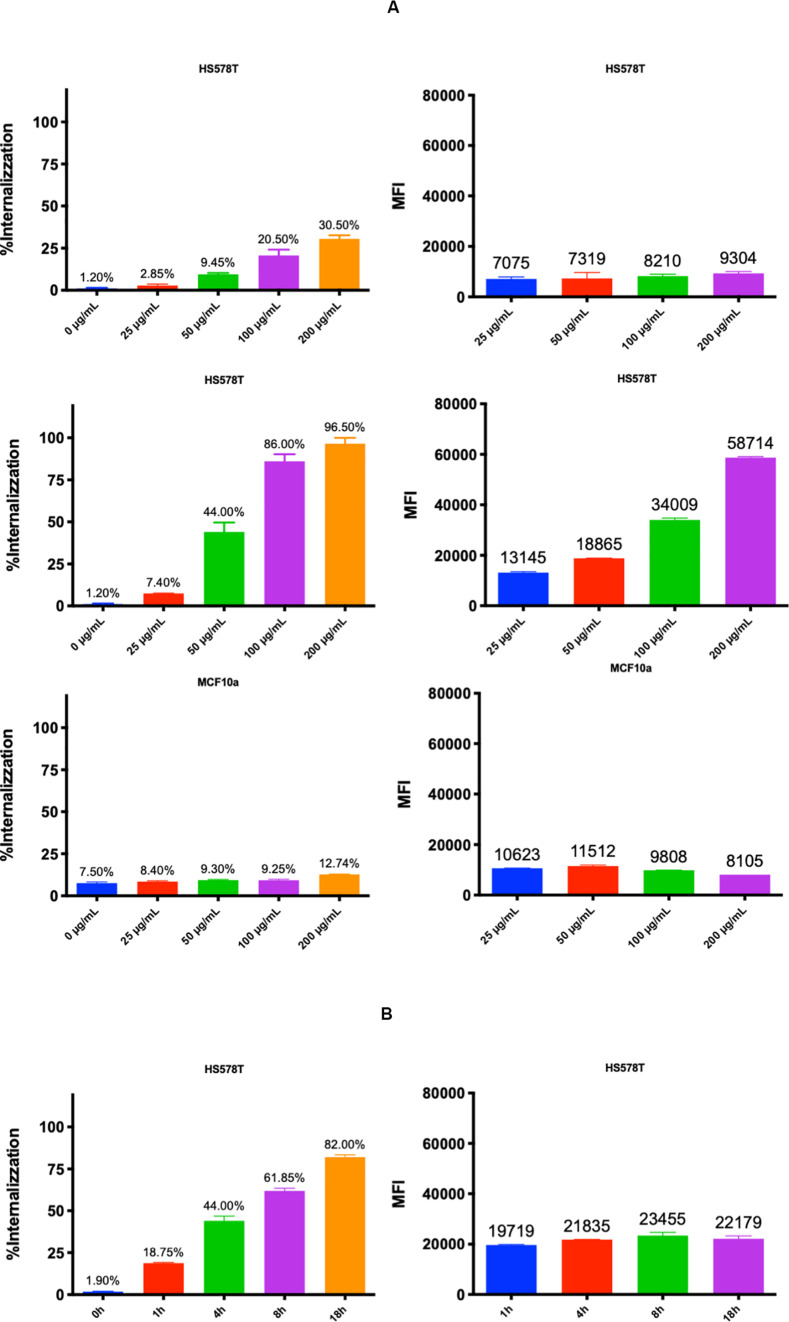
**(A)** Uptake of MelaSil_Ag* and MelaSil_Ag*-HSA NPs. Flow cytometry analysis of HS578T cells treated with bare and HSA-NPs for 18 h at increasing concentrations (upper and medium panel, respectively) and MCF10a cells treated with HSA-NPs in the same experimental conditions (lower panel). **(B)** Kinetic of MelaSil_Ag*-HSA NPs uptake. Flow cytometry analysis of HS578T cell lines treated with MelaSil_Ag*-HSA NPs for 1, 4, 8, and 18 h with 100 μg/mL MelaSil_Ag*-HSA NPs. NPs, nanoparticles; HSA, human serum albumin; MFI, mean fluorescence intensity.

The uptake of MelaSil_Ag^∗^-HSA NPs by HS578T and MCF10a cell lines was investigated by using fluorescent rhodamine conjugated NPs by flow cytometry and confocal microscopy. For flow cytometer analysis, HS578T (SPARC positive) and MCF10a (SPARC negative) cells were incubated with nanoparticles at 25, 50, 100, and 200 μg/mL for 18 h at 37 °C (Figure 4A).

For kinetic internalization analysis, HS578T cells were incubated with MelaSil_Ag^∗^-HSA NPs at 100 μg/mL for different times (Figure 4B). The results showed a concentration and time dependent MelaSil_Ag^∗^-HSA NPs uptake in HS578T cells, whereas no significant levels of uptake in MCF10a cells were observed. Furthermore, HS578T were incubated with bare NPs at 25, 50, 100, and 200 μg/mL for 18 h at 37 °C (Figure 4A). In this case the resulting fluorescence indicated a limited uptake due, more likely, to caveolae-dependent endocytosis, which occurs when anionic nanoparticles interact with positive sites on membrane proteins (Bannunah et al., 2014).

**FIGURE 5:**
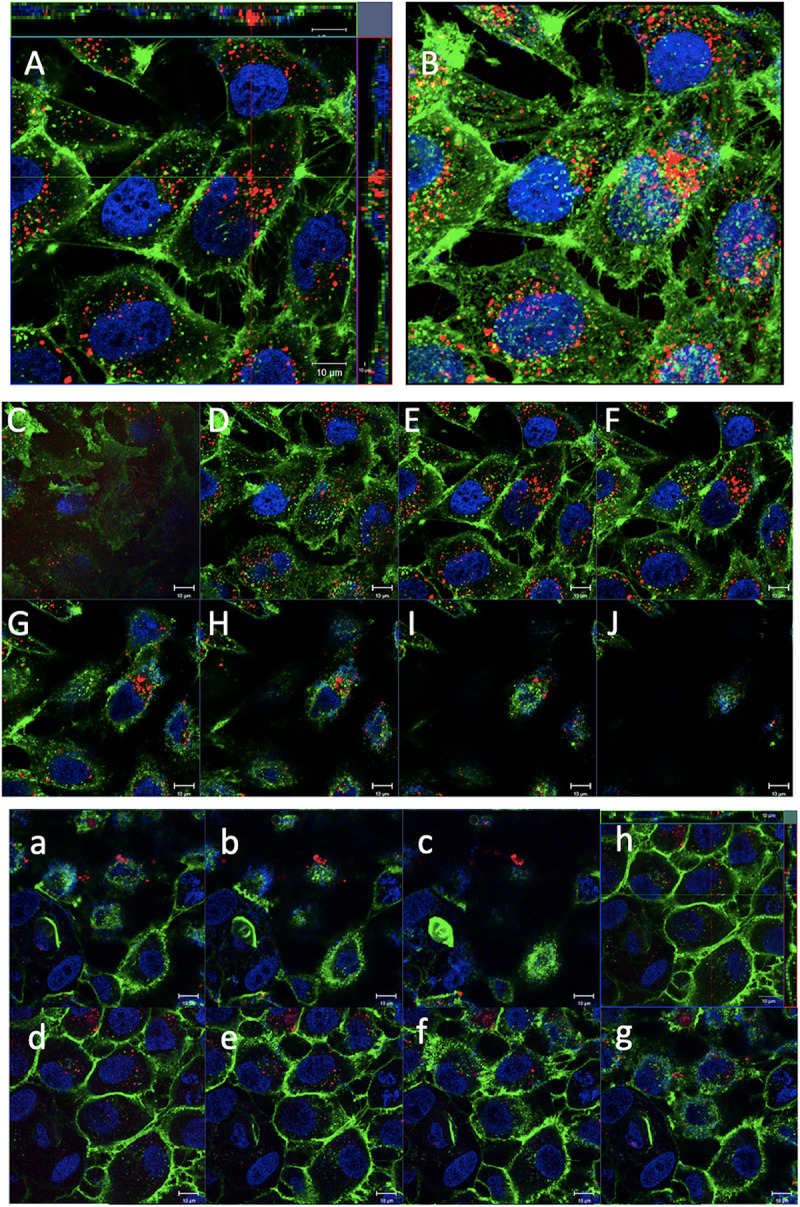
Confocal microscopy analysis of MelaSil_Ag*-HSA NPs internalization in HS578T and MCF10A cells. Representative images of confocal microscopy analysis of HS578T and MCF10a cell lines treated for 24 h with fluorescent MelaSil_Ag*-HSA NPs (50 μg/mL). **(A)** Orthogonal projection of z-stack acquisitions of HS578T cells treated with NPs and **(B)** 3D image reconstruction of Z-planes acquired from the top to the cells. **(C–J)** Gallery of merged images acquired along the *z*-axis of triple fluorescence-stained HS578T cells treated with NPs. **(a–g)** Gallery of merged images acquired along the z-axis of triple fluorescence-stained MCF10a cells treated with NPs. **(h)** Orthogonal projection of *z*-stack acquisitions of MCF10a cells treated with NPs. Cell nuclei and membranes were stained with Hoechst 33342 and WGA-Alexa Fluor 488, respectively. MelaSil_Ag*-HSA NPs is visible as red color. Scale bars: 10 μm. NPs, nanoparticles; HSA, human serum albumin.

For confocal microscopy analysis, both cell lines were incubated with MelaSil_Ag^∗^-HSA NPs for 18 h at 50 μg/ml (Figure 5). The confocal microscopy images showed the presence of nanoparticles as punctate small vesicles distributed in the cytoplasm of HS578T, suggesting an endocytic mechanism for the NPs internalization (Bannunah et al., 2014; Martucci et al., 2016). Furthermore, they confirmed the absence of MelaSil_Ag^∗^-HSA NPs in MCF10a cells.

### SPARC-HSA Interaction and Internalization Pathway of MelaSil_Ag^∗^-HSA Nanoparticles

As well known, SPARC, owing to its albumin binding properties, could promote the accumulation of albumin in cancer cells and tissues (Tai and Tang, 2008; Bannunah et al., 2014; Bairagi et al., 2015; Cheng et al., 2017; Parodi et al., 2019).

**FIGURE 6:**
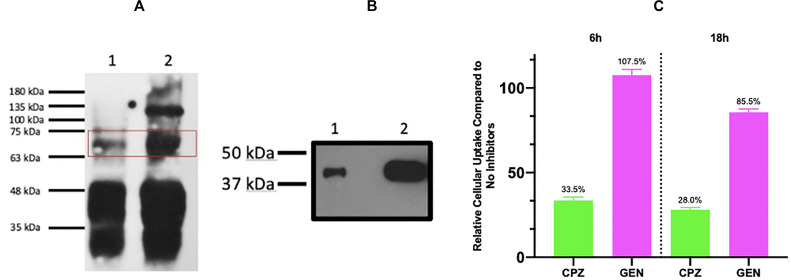
**(A)** Interaction between rHSA and SPARC. SPARC was immunoprecipitated and analyzed with an anti HSA antibody. HS578T cells extract with 10 μg (lane 1) and 50 μg (lane 2) of rHSA. **(B)** Interaction between MelaSil_Ag-HSA NPs and SPARC. MelaSil_Ag-HSA NPs-SPARC interaction (lane 1) and HS578T conditioned medium as positive control (lane 2). **(C)** Cytofluorimetric analysis of MelaSil_Ag*-HSA NPs internalization in HS578T after treatment with clathrin and caveolin inhibitors. NPs, nanoparticles; HSA, human serum albumin; CPZ, chlorpromazine; GEN, genistein, rHSA, recombinant HSA.

To evaluate the uptake mechanism of MelaSil_Ag^∗^-HSA NPs by HS578T, the interaction between SPARC and HSA was evaluated by a co-immunoprecipitation assay. To this aim, rHSA was added to HS578T cellular extract, co-immunoprecipitated with an anti-SPARC antibody and incubated with an anti-HSA antibody. Results clearly showed a chemiluminescent signal at about 65 kDa corresponding to albumin, indicating the interaction between the two proteins (Figure 6A). Furthermore, the binding of SPARC to the rHSA modified NPs surface, was evaluated. For this purpose, functionalized NPs were incubated in HS578T conditioned medium for 24 h. MelaSil_Ag-HSA NPs interacting proteins were resolved by SDS-PAGE and incubated with an anti-SPARC primary antibody. Results showed a chemiluminescent signal at about 43 kDa, proving the interaction of SPARC with MelaSil_Ag-HSA NPs surface (Figure 6B).

**FIGURE 7:**
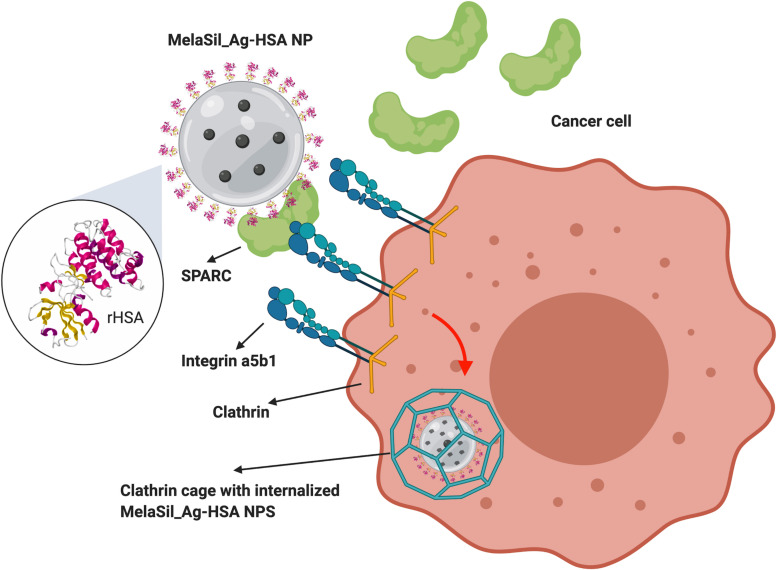
MelaSil_Ag-HSA internalization scheme. NPs, nanoparticles; HSA, human serum albumin.

Successively, to evaluate the uptake mechanism of MelaSil_Ag^∗^-HSA NPs by HS578T, cytofluorimetric analysis of cells incubated with nanoparticles at 100 μg/mL for 6 and 18 h, in absence or in presence of clathrin (chlorpromazine, 20 μg/mL) and caveolin (genistein, 80 μg/mL) inhibitors was performed (Figure 6C). Results showed a significant decrease of rHSA functionalized NPs internalization in cells treated with clathrin inhibitor, while no significant variation of internalization was observed in cells treated with caveolin inhibitor, suggesting a minor involvement of the caveolin pathway. As already reported, SPARC internalization is integrin-α5-dependent and it is regulated by a clathrin-mediated endocytosis (Nie et al., 2008; Nakamura et al., 2014; Hoang et al., 2015). Therefore, obtained data, together with literature data, could suggest an HSA-NPs SPARC-mediated internalization as shown in Figure 7.

### PA Signal of MelaSil_Ag-HSA in Cells, *ex vivo* and *in vitro* Test-Objects

**FIGURE 8:**
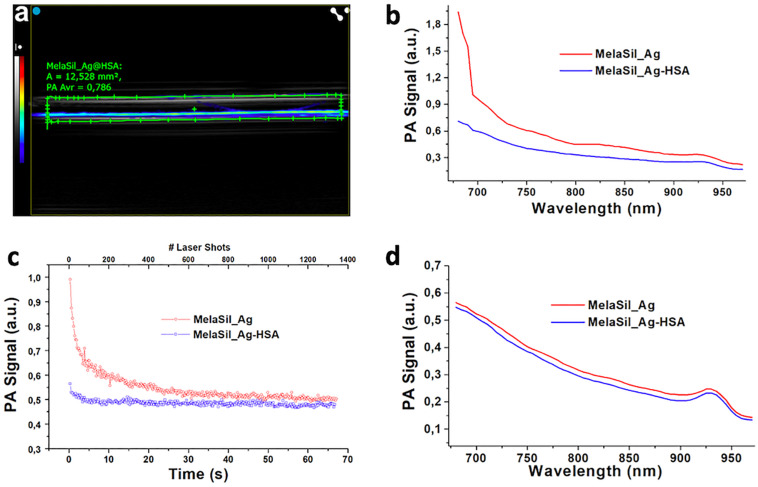
*In vitro* photoacoustic characterization of MelaSil_Ag-HSA NPs: **(a)** reference PA image of PE tubes loaded with MelaSil_Ag-HSA NPs, gray-bar for US signal, colored bar for PA; **(b)** PA Spectrum of MelaSil_Ag and MelaSil_Ag-HSA NPs before prolonged laser illumination; **(c)** Photostability over time at fixed wavelength of stimulation; **(d)** PA Spectrum of MelaSil_Ag and MelaSil_Ag-HSA NPs after prolonged laser illumination. NPs, nanoparticles; HSA, human serum albumin; PA, photoacoustic; PE, polyethylene, US, ultrasound.

**FIGURE 9:**
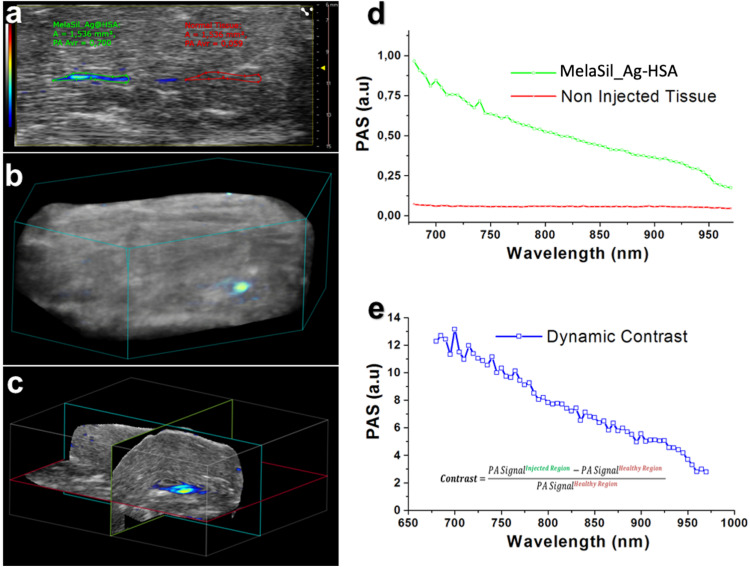
*Ex vivo* photoacoustic characterization of MelaSil_Ag-HSA NPs: **(a)** PA-US image of sample slice, gray-bar for US signal, colored bar for PA; **(b)** 3D PA-US volumetric reconstruction of chicken breast injected with MelaSil_Ag-HSA NPs (colored scale for PA Signal); **(c)** Cross View of PA-US planes of acquisition, where the PA signal showed the maximum intensity; **(d)** PA spectra provided from the drawn ROIs **(a)**; **(e)** Calculation of contrast values obtained from the injected region (5 mm depth) using the showed formulation. NPs, nanoparticles; HSA, human serum albumin; PA, photoacoustic; US, ultrasound, PE, polyethylene, ROI, region of interest.

**FIGURE 10:**
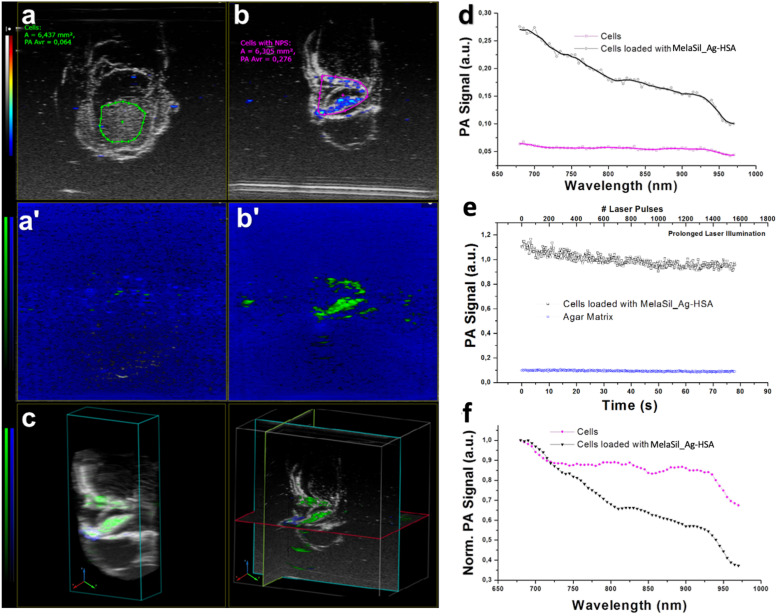
PA imaging of NPs loaded cells: **(a)** PA-US image of agar phantom with cells; **(b)** PA-US image of agar phantom with cells loaded with MelaSil_Ag-HSA NPs, grayscale for US, colored scale for PA; **(a’)**, **(b’)** previous PA images processed with spectral unmixing algorithm, green-bar for PA spectral signal from cells loaded with MelaSil_Ag-HSA NPs, blue-bar for PA spectral signal from the agar matrix; **(c)** on the left 3D PA-US volumetric reconstruction of a sample slice, on the right the cross viewing; **(d)** PA Spectra of loaded and control cells; **(e)** Photostability under prolonged laser illumination at 705 nm of charged and un-charged cells; **(f)** normalized spectra of charged and control samples. NPs, nanoparticles; HSA, human serum albumin; PA, photoacoustic; US, ultrasound.

MelaSil_Ag-HSA and MelaSil_Ag NPs were studied in PE tubes (Figure 8) as previously reported (Silvestri et al., 2019), showing the co-registered PA and ultrasound (US) signal: MelaSil_Ag-HSA NPs were more stable in the first part of optical windows of laser stimulation, as confirmed from the photostability tests. The two NPs solutions were stimulated over time (more than 1 min at 20 Hz laser pulse rate, over 1000 laser shots) at 710 nm, showing a standard variation of PA signal around 0.06 for the MelaSil_Ag and 0.01 for MelaSil_Ag-HSA NPs, leading to a signal to noise ratio (SNR) values of the order of 9 and 44, respectively. A bolus of 50 μL of MelaSil_Ag-HSA NPs was injected in a chicken breast tissue, used as biological matrix for PA (Figure 9). As reported in the 3D PA-US volumetric reconstruction and the cross view of PA-US acquisition planes (Figures 9b,c,d), the nanoparticles were clearly detectable and distributed more than 5 mm depth. The PA spectrum confirmed their typical spectral fingerprint (Figure 9e). The PA signals originating from NPs internalized by cells were tested and compared with control samples to demonstrate the significant difference in terms of PA response. Indeed, as reported in Figure 10, the PA signal provided by internalized NPs revealed a PA signal stronger compared to NPs free cells, with a spectral trend perfectly paring with MelaSil_Ag-HSA NPs. To better show the difference and detectability of NPs inside cells, spectral *unmixing* algorithms were applied to PA images (Figures 10a,b), fixing identical parameters (i.e., brightness, contrast and priority) for the processed images (Figures 10a’,b’): PA spectral trends were recorded for agar matrix (blue) and cells with NPs (green), respectively, and in panel c was reported the 3D PA-US volumetric reconstruction of a sample slice (left) with the related cross view (on the right). The intensity of PA after NPs uptake showed values up to four-time stronger compared to the NPs free cells (Figure 10d), with a maximum at 705 nm. The photostability was checked at 705 nm (Figure 10e), shooting the sample for over 1400 laser pulses (over 1 min with 20 Hz laser pulse rate 7). The PA signal was stable with a standard deviation around 0.05 a.u., showing a good SNR value around 20 and a contrast to noise ratio (CNR) value around 17. The PA normalized spectra, reported in panel f, underlined the capability of MelaSil_Ag-HSA NPs to be recognized during their use, indeed their specific spectral fingerprint allowed the mapping of distribution. While the PA spectrum of the cells without nanoparticles responded like a plateau trend in the range between 725 and 950 nm, the PA spectral trend of the internalized NPs showed a really different slope; in terms of absolute value, the slope of NPs inside cells showed a value six times higher. This plot showed as under around 725 nm the shapes were similar, but in this range the PA signal intensity of internalized NPs was more than four times higher compared to control. The presence of nanoparticles inside the cells also got an improvement of photostability as showed in Supplementary Figure S6.

## Conclusion

A tumor targeting nanoplatform displaying contrast functions through photoacoustic imaging was developed by functionalizing NPs surface with human serum albumin. Specific internalization was demonstrated *in vitro* by flow cytometry, in absence or presence of specific endocytosis inhibitors. Cytotoxicity tests demonstrated that MelaSil_Ag-HSA NPs concentrations ranging from 25 to 100 μg/mL have a negligible influence on cell viability and do not show hemotoxicity. Furthermore, the presence of HSA on the NPs surface reduced the nonspecific interactions of serum proteins that could affect NPs properties and behavior.

The PAI study demonstrated the increase of the photostability of MelaSil_Ag-HSA NPs compared to unmodified NPs, with a typical gain of one order of magnitude in terms of value of standard deviation under prolonged laser illumination. This allows to get suitable SNR and CNR, parameters really important for getting high quality and clear images during the acquisitions. In addition, MelaSil_Ag-HSA NPs may show promising photothermal effects, thus providing novel prototypes for multimodal imaging nanoplatforms with a potential use in theranostics, through hyperthermic effects caused by continuous wavelength laser stimulation. Pulsed lasers can modify or destroy the architecture of nanoparticles during illumination (reshaping phenomena), whereby the high photostability of MelaSil_Ag-HSA is of considerable relevance to ensure the same PA signal response *in vivo* for developing in loco treatment triggered by laser illumination.

## Data Availability Statement

The raw data supporting the conclusions of this article will be made available by the authors, without undue reservation.

## Author Contributions

AL and GS conceived the research. AL, LM, GL, AP, and MD’I supervised the overall work. BS and GP, prepared and characterized by dynamic light scattering and transmission electron microscopy, and the nanoparticles. GS performed the nanoparticles surface modification, *in vitro* cell culture studies including cellular uptake, cytotoxicity, hemotoxicity, and biochemical assessment. BC assisted GS in cell culture experiments and analysis. GC performed the confocal microscopy. PA performed all photoacoustic measurements. All authors discussed the results and contributed to the final manuscript.

## Conflict of Interest

The authors declare that the research was conducted in the absence of any commercial or financial relationships that could be construed as a potential conflict of interest.
