# Supplementary material for: Albumin-Modified Melanin-Silica Hybrid Nanoparticles Target Breast Cancer Cells via a SPARC-Dependent Mechanism
Source: Front Bioeng Biotechnol. 2020 Jul 8;8:765. doi: 10.3389/fbioe.2020.00765 (PMC7360861; doi:10.3389/fbioe.2020.00765)
Supplement: Supplementary file 1 [file Table_1.DOCX]

Supplementary Material

|  | **Fluorescence intensity Ex366 nm/Em 488 nm (A.U.)** |
| --- | --- |
| **MelaSil_Ag** | 8 |
| **MelaSil_Ag-APTES-NH_2_** | 40 |
| **MelaSil_Ag-HSA** | 250 |

**Table S1** Fluorescamine assay performed in HSA functionalization steps


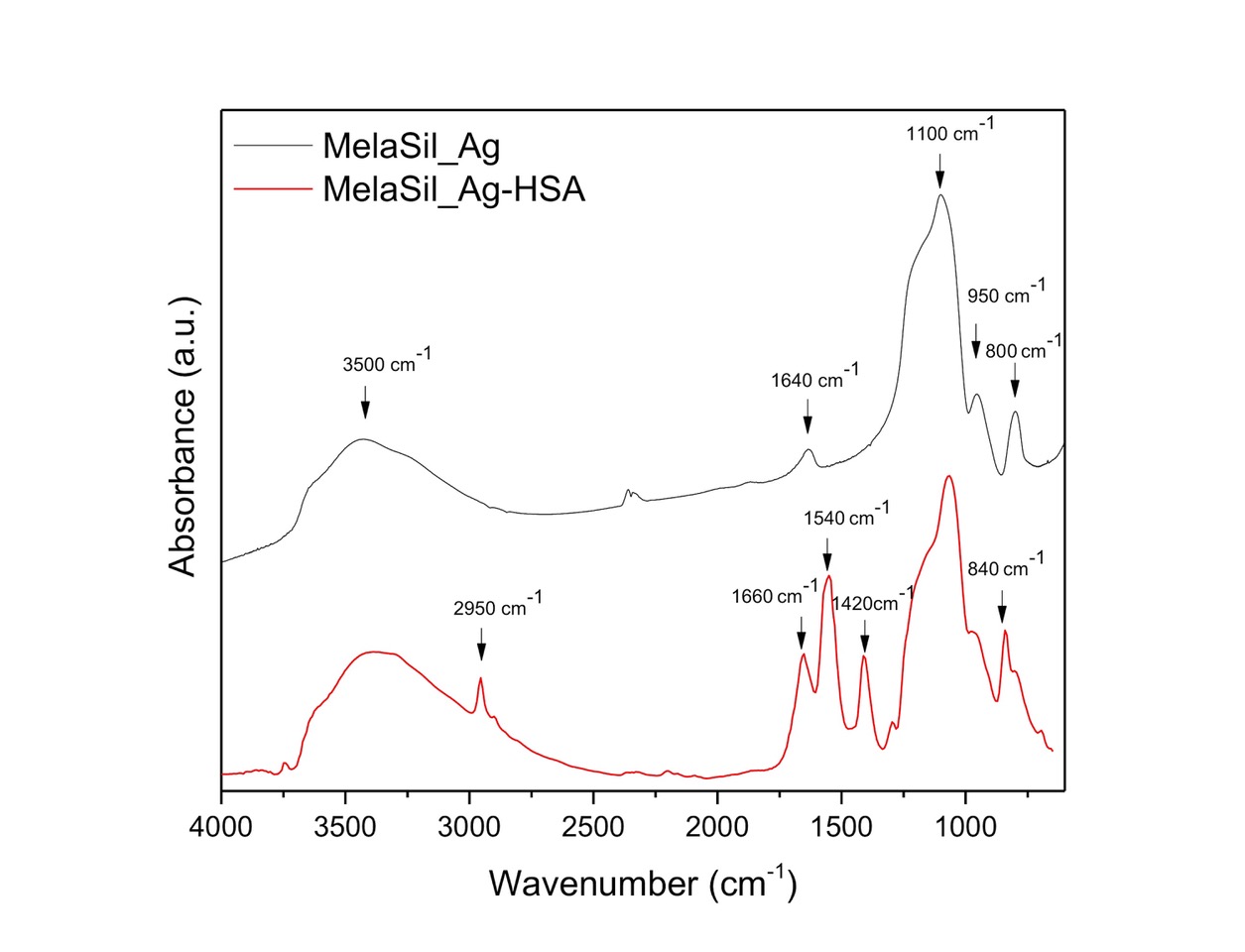


**Figure S1** FTIR spectra of bare and HSA-modified MelaSil_Ag NPs.

#
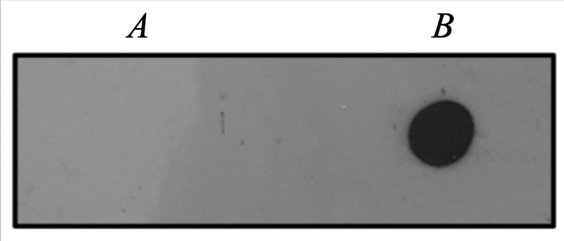


**Figure S2** Dot blot of MelaSil_Ag and MelaSil_Ag-HSA NPs. Dot blot performed with anti-HSA antibody on bare **(A)** and HSA functionalized NPs **(B)**. Abbreviations: NPs: Nanoparticles, HSA: Human Serum Albumin; NPs: Nanoparticles


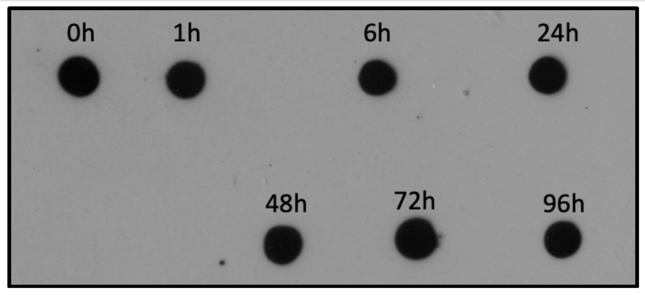


**Figure S3** Dot blot of MelaSil_Ag-HSA NPs in cell culture medium for different times. Abbreviations: HSA: Human Serum Albumin; NPs: Nanoparticles


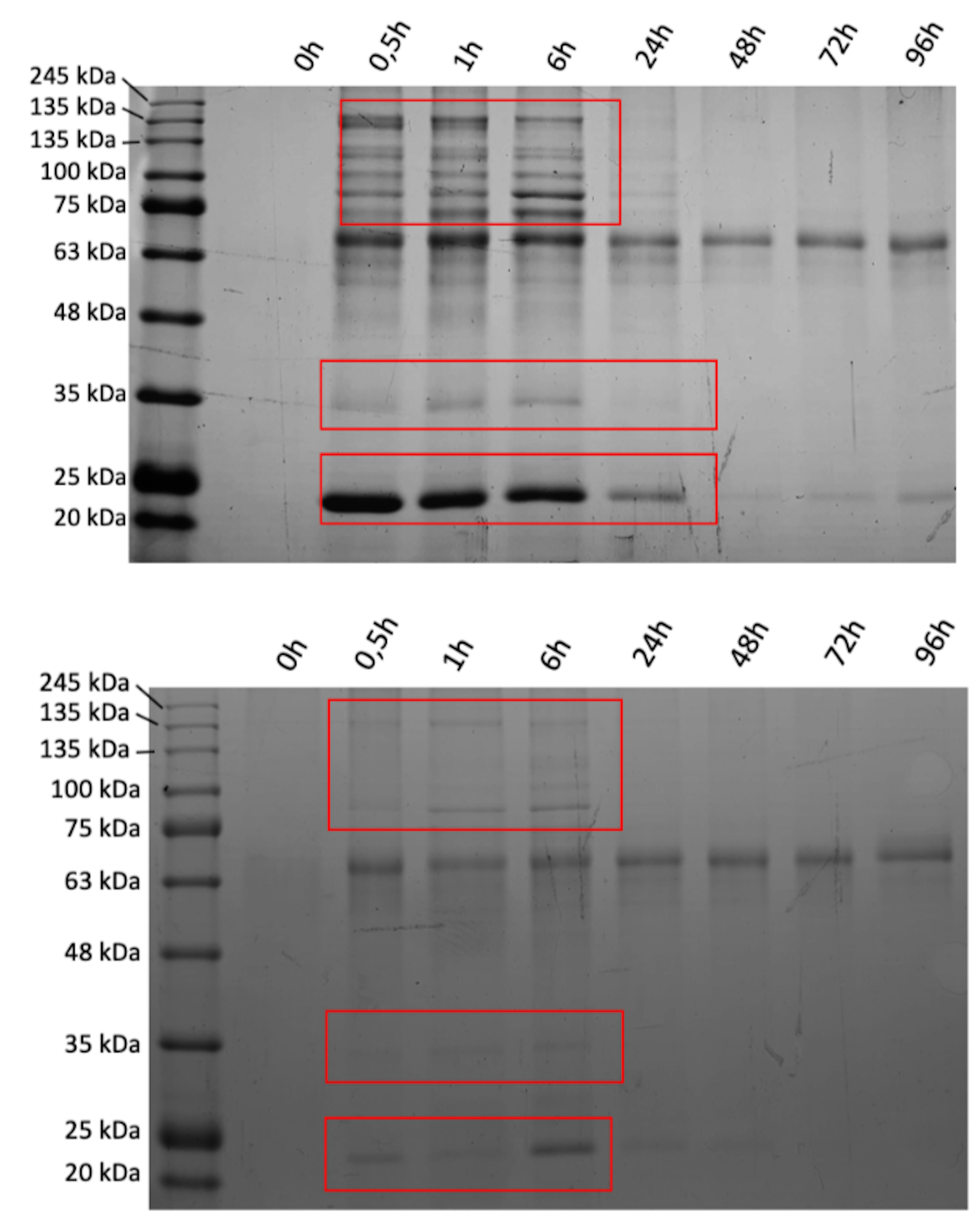


**Figure S4** SDS_PAGE of proteins adsorbed on MelaSil_Ag **(upper panel)** and MelaSil_Ag-HSA **(lower panel)** NPs surface. Abbreviations: NPs: Nanoparticles, HSA: Human Serum Albumin


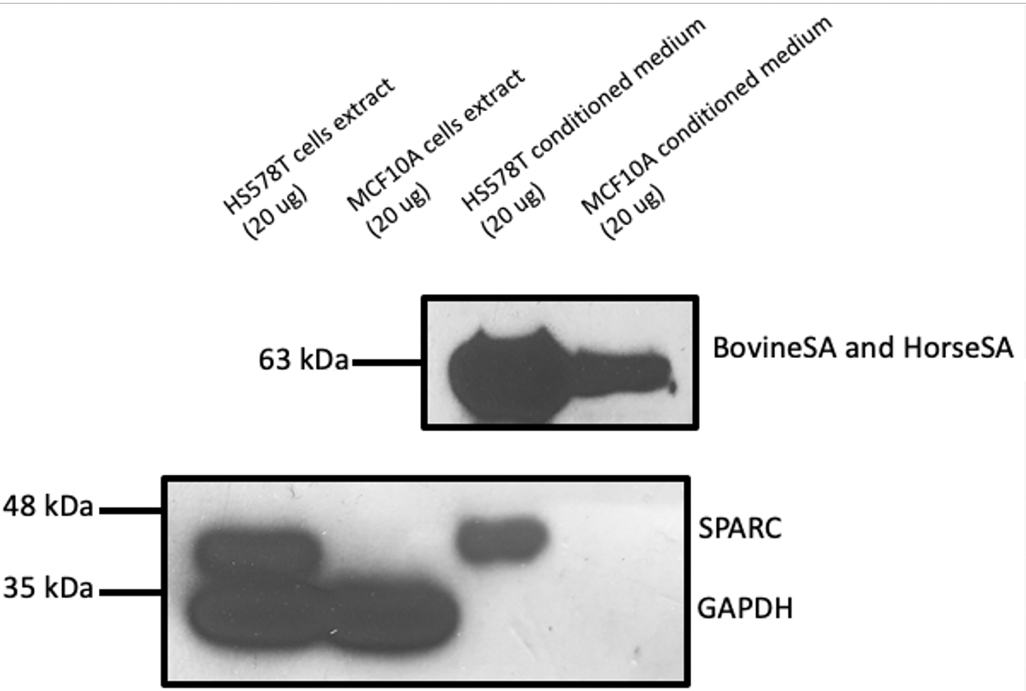


**Figure S5** Western Blot analysis. SPARC expression levels in HS578T and MCF10a whole cells extract and conditioned medium. Blot was performed with anti-SPARC antibody for all samples. For whole cells extract, anti-GAPDH antibody was used. For conditioned medium anti-BSA antibody was used.


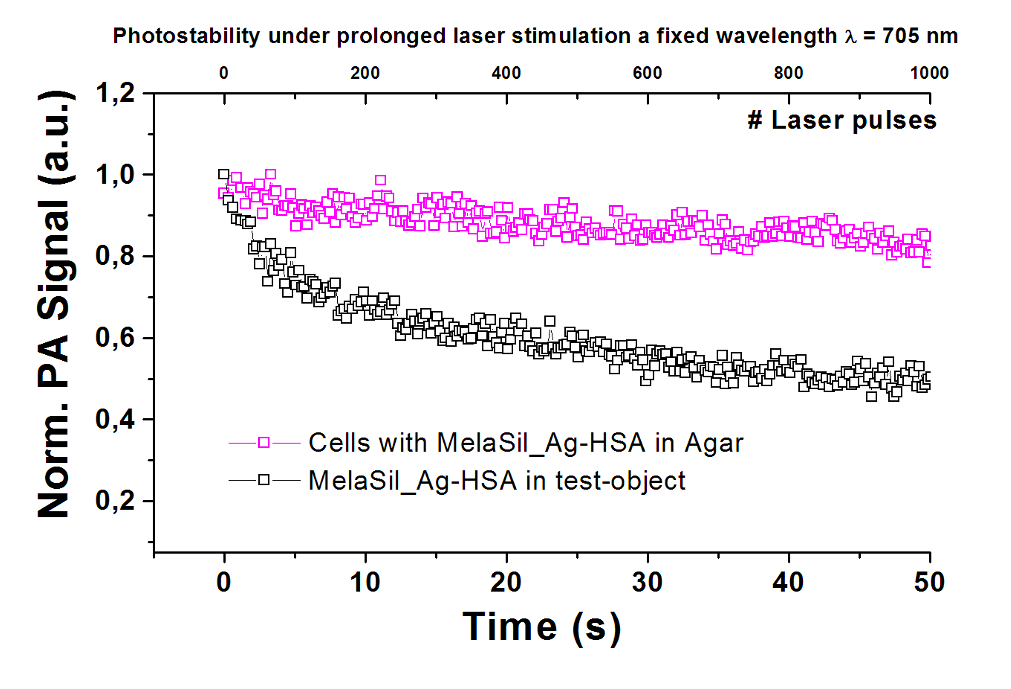


**Figure S6** The plot reports the photostability comparison between the normalized PA signals acquired from the MelaSil_Ag-HSA (*in vitro* test object) and that from the cells with the internalized NPs. Abbreviations: NPs: Nanoparticles, HSA: Human Serum Albumin, PA: Photoacoustic.
